# N-terminal Myristoylation Enhanced the Antimicrobial Activity of Antimicrobial Peptide PMAP-36PW

**DOI:** 10.3389/fcimb.2020.00450

**Published:** 2020-08-27

**Authors:** Yongqing Liu, Shengnan Li, Tengfei Shen, Liangliang Chen, Jiangfei Zhou, Shuaibing Shi, Yang Wang, Zhanqin Zhao, Chengshui Liao, Chen Wang

**Affiliations:** ^1^The Key Lab of Veterinary Biological Products, Henan University of Science and Technology, Luoyang, China; ^2^Department of Animal Science, University of Manitoba, Winnipeg, MB, Canada; ^3^Henan Provincial Open Laboratory of Key Disciplines in Environment and Animal Products Safety, Henan University of Science and Technology, Luoyang, China

**Keywords:** myristoylation, antibacterial peptide PMAP-36PW, antibacterial peptide Myr-36PW, antibacterial activity, stability, anti-biofilm, mechanism, therapeutic efficacy

## Abstract

Drug-resistant bacteria infections and drug residues have been increasing and causing antibiotic resistance and public health threats worldwide. Antimicrobial peptides (AMPs) are novel antimicrobial drugs with the potential to solve these problems. Here, a peptide based on our previously studied peptide PMAP-36PW was designed via N-terminal myristoylation and referred to as Myr-36PW. The fatty acid modification provided the as-prepared peptide with good stability and higher antimicrobial activity compared with PMAP-36PW *in vitro*. Moreover, Myr-36PW exhibited effective anti-biofilm activity against Gram-negative bacteria and may kill bacteria by improving the permeability of their membranes. In addition, the designed peptide Myr-36PW could inhibit the bacterial growth of *Staphylococcus aureus* ATCC 25923 and *Pseudomonas aeruginosa* GIM 1.551 to target organs, decrease the inflammatory damage, show an impressive therapeutic effect on mouse pneumonia and peritonitis experiments, and promote abscess reduction and wound healing in infected mice. These results reveal that Myr-36PW is a promising antimicrobial agent against bacterial infections.

## Introduction

Traditional antibiotic resistance and veterinary drug residues have been annually increasing and causing a serious global public health threat (CiŽman and Plankar Srovin, 2018). The World Health Organization (WHO) has emphasized that the incidence of infections caused by drug-resistant bacteria has been increasing globally and may kill 10 million people by 2050 (Woolhouse et al., 2016; Zhen et al., 2019). Antimicrobial peptides (AMPs) are new antimicrobial drugs (Mwangi et al., 2019a; Borro et al., 2020), particularly natural AMPs that have attracted attention due to their broad spectrum activity, rapid killing effect, and low induced resistance (Agarwal et al., 2016; Deslouches and Di, 2017). However, only few AMPs are currently in clinical application due to their instability, high cytotoxicity and low antimicrobial activity (Greber and Dawgul, 2016; Jacob et al., 2016). Many methods such as substitution (Zhou et al., 2019a), cyclization (Mwangi et al., 2019b), and hybridization (Miao et al., 2020) have been studied and adopted to overcome these shortcomings.

PMAP-36 is a typical amphiphilic α-helical antimicrobial peptide isolated from porcine myeloid cells (Lv et al., 2014). We previously designed PMAP-36PW using Trp (W) to replace the positions of 25 and 26 Pro (P) of PMAP-36, to increase hydrophobicity of PMAP-36 and provided it with high antibacterial activity, good stability, and effective reduction in bacterial load and tissue damage in infected mice (Zhou et al., 2019b). Myristic acid is a 14-carbon saturated fatty acid, and its modification is widely used in proteins and directs the cellular localizations (Martin et al., 2010). The antibacterial activity of antimicrobial peptides can be improved by N-terminal myristoylation, and N-terminal myristic acid modification is a simple and feasible treatment strategy for drug-resistant bacterial infections (Krishnakumari et al., 2018; Lei et al., 2018).

In this study, an N-terminal myristoylated antimicrobial peptide Myr-36PW was designed to improve antimicrobial activities. A series of experimental methods was performed to investigate the antibacterial activity, stability, toxicity, anti-biofilm activity, and antibacterial mechanism *in vitro* and therapeutic effect in mice infected with *Staphylococcus aureus* ATCC 25923 and *Pseudomonas aeruginosa* GIM 1.551.

## Materials and Methods

### Bacterial Strain, Antibiotic, Reagents, and Mice

*S. aureus* ATCC 25923 were kindly provided by Bin Tang at Jilin University (China). *Listeria monocytogenes* CICC 21634 was isolated from clinical cases and maintained in the China Center of Industrial Culture Collection (CICC, China). *Salmonella typhimurium* SL 1344 and *P. aeruginosa* GIM 1.551 specimens were isolated from clinical cases and maintained in our laboratory. NIH 3T3 cells were kindly provided by Dr. Lu at Nanjing Medical University (China). Dulbecco's-modified eagle medium (DMEM) and neonatal bovine serum (NBS) were purchased from Sijiqing Biotech (China). Ceftiofur sodium, ampicillin sodium, benzylpenicillin potassium, lysogenic broth (LB) medium, trypticase soy broth (TSB) medium, brain heart infusion (BHI) medium, Mueller-Hinton broth (MHB) medium, mice serum, Triton X-100, methyl thiazolyl diphenyltetrazolium bromide (MTT), dimethyl sulfoxide (DMSO) and propidium iodide (PI) were purchased from Procell Corporation (Wuhan, China). A total of 360 specific pathogen-free (SPF) BALB/c mice (aged 4–6 weeks, weighting 20 ± 3 g, with equal numbers of males and females) were purchased from Henan Province Experimental Animal Center (Henan, Chain). All animal experiments were conducted in accordance with the guidelines and with the approval of the Animal Experiment Committee of Henan University of Science and Technology (No. 20190719001).

### Peptide Synthesis

PMAP-36, PMAP-36PW, and Myr-36PW were synthesized by Fmoc-chemistry at Shanghai Apeptide Biological Technology Co., Ltd (Shanghai, China). All peptides were purified by reverse phase high-performance liquid chromatography (HPLC) to a purity of >95%, and the synthesized peptides were identified by using electrospray ionization mass spectrometry (MS).

### Inhibition Zone Assay

The inhibition zone assay was performed through the disk diffusion to evaluate the antimicrobial activity of Myr-36PW *in vitro* (Dunne et al., 2017). The four above-mentioned bacterial strains were used as experimental strains, with *L. monocytogenes* CICC 21634 cultured in BHI medium, and *P. aeruginosa* GIM 1.551 cultured in TSB medium. The bacterial cells were cultured for 12 h at 37°C in the appropriate medium, and the bacterial cell suspension was spread on LB agar. The disks containing 10 μg of peptides (1 μg/μL) were attached to the solid medium and incubated at 37°C for 18 h. Finally the diameter of the inhibition zone was measured.

### MIC Assay

The MICs of Myr-36PW *in vitro* were measured through microdilution in accordance with the Clinical and Laboratory Standards Institute (CLSI, 2020). The bacterial cells were cultured for 12 h at 37°C in the appropriate medium and then diluted to 1 × 10^6^ CFU/mL. Equal volumes (100 μL) of bacterial cell suspension and two-fold serially diluted different concentrations peptides (0.0039–256 μg/mL) were added to each well of the sterile 96-well plate. The samples were incubated for 18 h at 37°C. After incubation, the MICs of the peptides were determined by evaluating the growth based on the OD_600_ of the cultures.

### Thermal Stability Testing

For thermal stability analysis, the Myr-36PW and peptides were boiled for 10, 20, 30, 40, 50, 60, 90, and 120 min as reported (Ebbensgaard et al., 2015) with unboiled peptides as controls. Inhibition zone assay was performed against *S. aureus* ATCC 25923 and *P. aeruginosa* GIM 1.551. The inhibition zone diameters of peptides (10 μg, 1 μg/μL) were measured after incubation for 18 h at 37°C, and the inhibition curve was drawn.

### pH Stability Testing

For stability test, the Myr-36PW and peptides were treated with pH 2–13, and PBS buffer with different pH values was used as a control (Wang et al., 2019). Inhibition zone assay was performed against *S. aureus* ATCC 25923 and *P. aeruginosa* GIM 1.551. The inhibition zone diameters of peptides (10 μg, 1 μg/μL) were measured after incubation for 18 h at 37°C, and the inhibition curve was drawn.

### Salt and Serum Stability Assays

The effects of abiotic factors on the antibacterial activity of Myr-36PW were investigated by MIC assay (Xi et al., 2013; Zhong et al., 2019). *S. aureus* ATCC 25923 and *P. aeruginosa* GIM 1.551 overnight cultures were diluted to 1 × 10^6^ CFU/mL, and the MICs of peptides in MHB with different concentration of physiological salts (150 mM NaCL, 2 mM CaCL_2_) were determined. And after the peptides incubated in 10% mice serum for 1 h at 37°C, MICs of peptides against *S. aureus* ATCC 25923 and *P. aeruginosa* GIM 1.551 were determined. The MICs in MHB in the absence of physiological salts and serum were used as the control group.

### Hemolysis Assay

The *in vitro* hemolytic activity of Myr-36PW was evaluated as described previously (Singh et al., 2016). In brief, 100 μL mouse erythrocyte suspension (final concentration 8% v/v) was treated with 100 μL of peptides (2.5–640 μg/mL) in a 96-well plate incubated for 1 h at 37°C, and then centrifuged at 1,000 × g for 5 min. The supernatant was transferred to another 96-well plate, and the absorbance was measured at 570 nm. PBS and 0.2% Triton X-100 served as the negative and positive controls, respectively. Hemolysis rate (%) = [OD_570 nm(peptides)_ – OD_570 nm(PBS)_]/[OD_570 nm(TritonX−100)_ – OD_570 nm(PBS)_] × 100%.

### Cytotoxicity Assay

NIH 3T3 cells were used to evaluate the cytotoxicity of Myr-36PW *in vitro* by MTT assays (Jia et al., 2019). The NIH 3T3 cells were cultured in DMEM with 10% NBS and seeded in 96-well plate (2 × 10^5^cells/well). After incubation for 24 h at 37°C, different concentrations of peptides (2.5–640 μg/mL) were added to the wells and incubated for 24 h at 37°C. Afterward, 20 μL MTT solution (5 mg/mL) was added to each well and incubated for another 4 h at 37°C. The medium was completely removed, and 150 μL of DMSO was added in each well to dissolve the formazan crystals. Finally, absorbance was measured at 570 nm.

### Biofilm Inhibition Assay

The biofilm-inhibiting ability of Myr-36PW was evaluated against *S. aureus* ATCC 25923, *S. typhimurium* SL 1344, and *P. aeruginosa* GIM 1.551 by using crystal violet dye (Chen et al., 2018; de Breij et al., 2018). Overnight cultured strains in TSB medium were diluted to 1 × 10^6^ CFU/mL, 100 μL of bacterial suspension with 100 μL of different concentration of peptides (0.25–128 μg/mL) was added into a 96-well plate. Bacterial suspension without peptides was used the negative control. After incubation for 24 h at 37°C, the supernatant weas discarded, and the biofilm was gently washed with PBS, fixed in methanol for 20 min, stained with 0.1% (w/v) crystal violet for 10 min, and finally removed. After washing with PBS, 95% ethanol was added into wells, and the optical density (OD) was measured at 620 nm.

### Biofilm Eradication Assay

The biofilm-eradicating ability of Myr-36PW was determined against *S. aureus* ATCC 25923, *S. typhimurium* SL 1344, and *P. aeruginosa* GIM 1.551 (Chen et al., 2018; de Breij et al., 2018). In brief, 200 μL of bacterial dilution (1 × 10^6^ CFU/mL) was added into a 96-well plate and incubated for 24 h at 37°C to form a biofilm, which was washed three times with PBS. Subsequently, 200 μL of peptides at 128 μg/mL were added to the wells. After incubation for 1 h at 37°C, the OD of each well was measured at 620 nm by crystal violet staining. The wells were gently washed three times with PBS after 1 h incubation to evaluate the killing effect of Myr-36PW against bacteria in the biofilm. Afterward, 100 μL of suspension was serially diluted and spread on LB agar plates, and bacterial colony was measured.

### Membrane Permeability Assay

The membrane permeability assay was performed to analyze the bacterial killing of Myr-36PW by using inverted fluorescence microscope with PI as a fluorescent indicator. Overnight cultured bacteria (1.5 mL) were centrifuged at 2,000 × g for 5 min and then washed three times with PBS. The bacterial precipitates were resuspended with peptides (1.5 mL) at a concentration of MIC (*P. aeruginosa* GIM 1.551), 2MIC (*S. typhimurium* SL 1344), and 4MIC (*S. aureus* ATCC 25923 and *L. monocytogenes* CICC 21634), and PBS was added as a control. The samples were incubated at 37°C for 30 min. Equal volumes of PI (200 μg/mL) were added and incubated at 37°C for 10 min in the dark environment. The samples were placed on a glass slide, covered with coverslip, and dried. Bacterial cells were imaged by an inverted fluorescence microscope.

### Experimental of Lung Infection and Treatment

A pneumonia model *in vivo* was established as previously reported, with minor modification (Cortés et al., 2002; Aoki et al., 2009; Shi et al., 2018). A total of 120 mice were randomly divided into two experimental models, one was intranasal inoculation of *S. aureus* ATCC 25923 (8.3 × 10^9^ CFU/mL, 50 μL), and the other was intranasal inoculation of *P. aeruginosa* GIM 1.551 (4.5 × 10^10^ CFU/mL, 50 μL). In the *S. aureus* ATCC 25923 model, 60 mice were divided into the following six groups of 10 mice each: no infection and treatment was used as a blank control, those with intranasal administration of PBS (40 μL) as the negative control group, those administered with benzylpenicillin potassium (40 μL, 1 μg/μL) as the positive control group, and the remaining three corresponded to Myr-36PW (40 μL, 1 μg/μL), PMAP-36PW (40 μL, 1 μg/μL), and PMAP-36 groups (40 μL, 1 μg/μL). The procedure for *P. aeruginosa* GIM 1.551 model is the same as for *S. aureus* ATCC 25923 model, except for the use of ampicillin sodium as the positive control. After 4 h post-inoculation, treatment was initiated, once a day for 3 days. The mice were sacrificed 24 h after the treatment. Changes in the appearance of lung tissues were observed, and lung tissues were removed aseptically from the mice. Half of the removed lung tissues were gently fixed in buffered formaldehyde solution for histopathological assessment by hematoxylin and eosin (H&E) staining. Pathological changes were scored to provide individual therapeutic status as described previously (Lei et al., 2018). The remaining lung tissues were added with 1 mL of PBS, homogenized, and serially diluted. Bacterial CFU counts were determined.

### Experimental of Peritonitis Model

The peritonitis model *in vivo* was generated by intraperitoneally injecting *S. aureus* ATCC 25923 (2.5 × 10^9^ CFU/mL, 140 μL). Sixty mice were randomly divided into six groups of 10 members: those who received no infection and treatment was used as a blank control, those with intranasal administration of PBS (60 μL) as the negative control group, those administered with benzylpenicillin potassium (60 μL, 1 μg/μL) as the positive control group, and the remaining three groups corresponded to three antimicrobial peptide groups of Myr-36PW (60 μL, 1 μg/μL), PMAP-36PW (60 μL, 1 μg/μL), and PMAP-36 (60 μL, 1 μg/μL). After 4 h post-inoculation, treatment was initiated by intraperitoneal injection once a day for 3 days. The mice were sacrificed 24 h the treatment. Changes in the appearance of anatomical tissues were observed. The liver and spleen tissues were removed aseptically from the mice, and half of these samples were gently fixed in buffered formaldehyde solution for histopathological assessment by hematoxylin and eosin (H&E) staining. Pathological changes were scored to assess individual therapeutic status as described previously (Song et al., 2015; Lei et al., 2018). The remaining half of livers and spleens were added with 1 mL of PBS, homogenized, and serially diluted. Bacterial CFU counts were measured.

### Experimental of Skin Infection and Treatment

A subcutaneous abscess model *in vivo* was established as previously reported (Zhao et al., 2019). The mouse dorsal region with 3.0 cm diameter was treated with hair pusher. A total of 120 mice were randomly divided into two experimental models, one was challenged with *S. aureus* ATCC 25923 (2.5 × 10^8^ CFU/mL, 60 μL), and the other was challenged with *P. aeruginosa* GIM 1.551 (3.0 × 10^8^ CFU/mL, 60 μL). In the *S. aureus* ATCC 25923 model, 60 mice were divided into six groups of 10 members. The mice with no infection and treatment was used as a blank control, those directly injected with PBS (20 μL) as the negative control group, those injected with benzylpenicillin potassium (20 μL, 1 μg/μL) as the positive control group, and the remaining three groups as the treatment groups injected with Myr-36PW (20 μL, 1 μg/μL), PMAP-36PW (20 μL, 1 μg/μL), and PMAP-36 (20 μL, 1 μg/μL). The procedure in *P. aeruginosa* GIM 1.551 model is the same as that in *S. aureus* ATCC 25923 model, except for the use of ampicillin sodium as a positive control. Treatment once a day for 3 days was initiated when the mice developed prominent pustules on their backs. A day after the treatment, the size of the abscess was observed, and its tissues were excised, homogenized, and serially diluted. Bacteria CFU counts were calculated.

### Experiment of Wound Infection and Treatment

Sixty mice with ten mice in each group were used in the experiment. Infection and treatment were performed as previously described (Hong et al., 2017). The mouse dorsal region with a diameter of 3.0 cm was treated with hair pusher, and a full-thickness 2.0 cm diameter wound was created by excising the skin and infected by direct seeding with *P. aeruginosa* GIM 1.551 (1 × 10^8^ CFU/mL, 50 μL). The wound was then covered with sterile gauze to prevent cross contamination. Treatment was initiated at 24 h after bacterial inoculation. The mice with no infection and treatment was used as a blank control, those provided with PBS (20 μL) as the negative control group, those administered with ampicillin sodium (20 μL, 1 μg/μL) as the positive control group, and the remaining were those treated with the three peptides (20 μL, 1 μg/μL) once a day for 7 days as the treatment groups. The mice were sacrificed after the 7 day treatment. Changes in the wound were observed, and the infected sections of skin were aseptically contained, homogenized, and serially diluted. Bacterial CFU counts were obtained.

### Statistical Analysis

Experimental data were encoded in Graphpad 8.0 and presented as mean ± SD. Statistical analyses were performed using unpaired *t*-tests or one-way ANOVA *F*-statistics, and differences were considered significant at *P* < 0.05 or *P* < 0.01. The sections were observed using Pannoramic Viewer software.

## Results

### Peptide Design and Characterization

**Figure 1:**
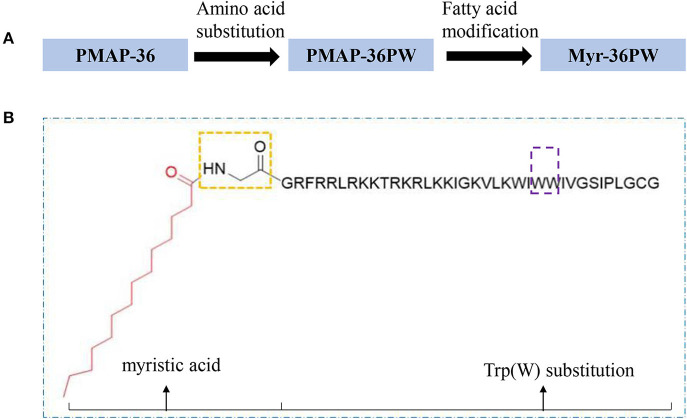
Peptides timeline and Myr-36PW molecular structure. **(A)** The timeline of peptide design from PMAP-36 to PMAP-36PW to Myr-36PW. **(B)** Myr-36PW molecular structure. The red structure indicates myristic acid, the yellow frame indicates an amide bond containing a Gly linker, the sequence represents PMAP-36PW, and the purple frame indicates Trp(W) substitution at positions 25 and 26 of PMAP-36. Myristic acid was tethered to N-terminal PMAP-36PW via the amide bond containing a Gly residue to form Myr-36PW.

**Figure 2:**
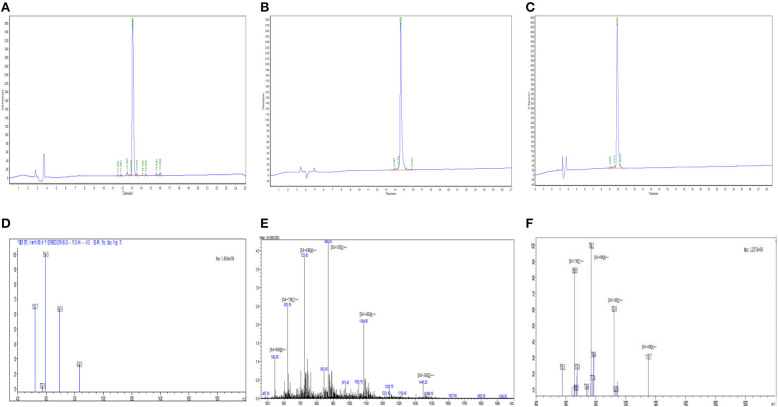
HPLC and mass spectrometry analysis of Myr-36PW and its analogs and Myr-36PW molecular structure. **(A–C)** HPLC of PMAP-36, PMAP-36PW, and Myr-36PW. Analytical HPLC was performed on a Waters 2690 Alliance system equipped with a Waters Gemini-NX C18 column (5 μm, 4.6 × 250 mm) at a flow rate of 1.0 mL/min using a gradient of 5–65% acetonitrile in water with 0.1% TFA as the mobile phase. UV detection was at a wavelength of 220 nm. **(D–F)** Mass spectrometry of PMAP-36, PMAP-36PW, and Myr-36PW. The source conditions were as follow: nebulizer gas flow: 1.5 L/min, CDL temperature: 250°C, capillary voltage: 1,500 V. T. flow: 0.2 ml/min. The mass scan was in the range of m/z 400–2,000.

Myr-36PW was designed through fatty acid modification to further improve the antibacterial activity of PMAP-36PW. Myristic acid, a 14-carbon saturated fatty acid was selected to couple with the N-terminus of PMAP-36PW via an amide bond containing a Gly linker, which aims to avoid the effect of fatty acids on the conformation of peptide. The synthesized peptides and their sequences and biochemical parameters are listed in Table 1, and the peptides timeline and molecular structure are shown in Figure 1. HPLC and MS results are shown in Figure 2. MS indicated that the molecular weights of PMAP-36 (4157.16), PMAP-36PW (4335.45), and Myr-36PW (4603.77) were nearly consistent with their theoretical molecular weights, indicating the successfully synthesis of the peptides. The overall hydrophobicity of the peptides was analyzed by HPLC and represented by their retention time: 13.080 min for PMAP-36, 14.628 min for PMAP-36PW, and that of Myr-36PW was prolonged. Therefore, Myr-36PW gained a fatty acid, and its hydrophobicity was enhanced.

**Table 1:** Amino acid sequences and biochemical parameters of Myr-36PW and its analogs.

**Peptide**	**Sequence**	**Theoretical MW**	**Measured MW**	**Net charge**	**T_**R**_(min)**	**Theoretical pI**
PMAP-36	GRFRRLRKKTRKRLKKIGKVLKWIPPIVGSIPLGCG	4157.22	4157.16	+13	13.080	12.31
PMAP-36PW	GRFRRLRKKTRKRLKKIGKVLKWIWWIVGSIPLGCG	4335.41	4335.45	+13	14.628	12.31
Myr-36PW	Myr-G-GRFRRLRKKTRKRLKKIGKVLKWIWWIVGSIPLGCG	4604.85	4603.77	-	16.458	-

*Molecular weight (MW) was measured by mass spectrometry*.

*Net charge was calculated by antimicrobial peptide database (http://aps.unmc.edu/AP/prediction/prediction_main.php)*.

*T_R_: retention time measured by analytical HPLC*.

*Theoretical pI was determined with https://web.expasy.org/cgibin/protparam/protparam*.

-, *indicated without testing*.

### Antimicrobial Activity of Myr-36PW *in vitro*

Two Gram-positive and two Gram-negative strains were used as test strains for inhibition zone and MIC assays to investigate the antimicrobial activity of Myr-36PW *in vitro*. Myr-36PW generally exhibited antimicrobial activity against the four bacterial strains, and the inhibition zones are shown in Supplementary Figure 1. Compared with PMAP-36PW, Myr-36PW displayed higher antimicrobial activity, whereas ceftiofur sodium showed no significant difference (Supplementary Table 1). The MICs of Myr-36PW ranged from 0.0020 to 8 μg/mL, which were four times higher than those of PMAP-36PW against *S. aureus* ATCC 25923 and *L. monocytogenes* CICC 21634 and two times higher than those of PMAP-36PW against *S. typhimurium* SL 1344 and *P. aeruginosa* GIM 1.551 (Table 2). In summary, the antimicrobial activity of N-terminal myristoylated Myr-36PW was higher than that of PMAP-36PW *in vitro*.

**Table 2:** MICs of Myr-36PW against bacteria.

**Bacteria strain**	**MICs (μg/mL)^&^**		
	**PMAP-36**	**PMAP-36PW**	**Myr-36PW**
*S. aureus* ATCC 25923	0.0156	0.0078	0.0020
*L. monocytogenes* CICC 21634	4	2	0.5
*S. typhimurium* SL 1344	32	16	8
*P. aeruginosa* GIM 1.551	1	0.5	0.25

& *MICs (Minimum inhibitory concentrations) were determined as the lowest concentration of the peptides that inhibited bacteria growth*.

### Stability of Myr-36PW Exposed to Heat, pH, Salt, and Serum

**Figure 3:**
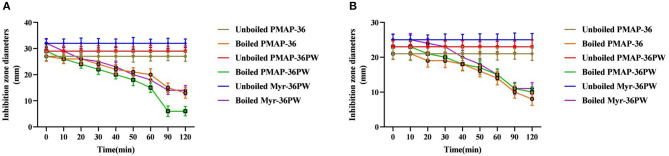
Thermal stability determination of Myr-36PW by boiling. Thermal stability test of Myr-36PW and its analogs by boiling in different times (0, 10, 20, 30, 40, 50, 60, 90, and 120 min) via disk diffusion method. **(A)** (*S. aureus* ATCC 25923) and **(B)** (*P. aeruginosa* GIM 1.551) show the thermal stability curves of PMAP-36, PMAP-36PW, and Myr-36PW. The diameter of the standard disk is 6 mm. Data represent the mean ± SD (*n* = 5).

**Figure 4:**
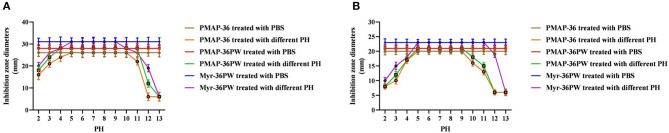
PH stability determination of Myr-36PW. PH stability test was performed using Myr-36PW and its analogs treated with different pH levels (2–13) through disk diffusion method. **(A)** (*S. aureus* ATCC 25923) and **(B)** (*P. aeruginosa* GIM 1.551) show the pH stability curve of PMAP-36, PMAP-36PW, and Myr-36PW. The diameter of the standard disk is 6 mm. Data represent the mean ± SD (*n* = 5).

Inhibition zone and MIC assays were performed to determine the effects of heat, pH, salt, and serum on Myr-36PW activity. The thermal stability results are shown in Figure 3. Boling reduced the antibacterial activity of Myr-36PW and its analogs against *S. aureus* ATCC 25923 and *P. aeruginosa* GIM 1.551. The activity of peptides continued to decrease with prolonged boiling. However, the antimicrobial activity of Myr-36PW was heat-stable, retaining high bacterial inhibition against *S. aureus* ATCC 25923 and *P. aeruginosa* GIM 1.551 even after boiling for 120 min. By contrast, PMAP-36 and PMAP-36PW showed low antimicrobial activity after boiling 120 min. In addition, Myr-36PW exhibited pH stability against *S. aureus* ATCC 25923 at pH 5–9 and *P. aeruginosa* GIM 1.551 at pH 5–11. Its antimicrobial activity was higher than that of PMAP-36PW at pH 2 and disappeared in pH 13. PMAP-36PW showed minimal or no antimicrobial activity at pH 12 (Figure 4). Supplementary Table 2 shows the effect of salt and serum on the antimicrobial activity of Myr-36PW. The MICs for all peptides were not changed compared with those in the absence of physiological NaCL, but those of Myr-36PW were changed in the presence of physiological CaCL_2_, about a half or a quarter of the MICs of the control group against *S. aureus* ATCC 25923 and twice that of the control group against *P. aeruginosa* GIM 1.551. PMAP-36PW displayed a quarter of control group against *S. aureus* ATCC 25923 and unchanged against *P. aeruginosa* GIM 1.551. After pre-incubation in 10% serum for 1 h, Myr-36PW showed a twice MIC of control group against two bacteria, the other peptides did not show any change in MIC. Although the MICs of Myr-36PW had changed, its antimicrobial activity still remained. These results revealed that Myr-36PW can resist physical and chemical challenges and maintain its good stability.

### Hemolysis and Cytotoxicity of Myr-36PW

**Figure 5:**
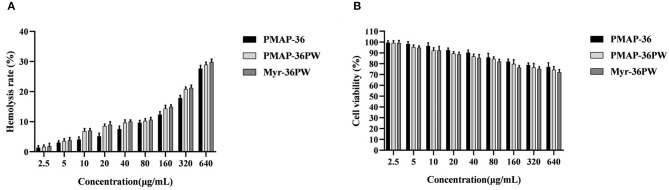
*In vitro* toxicity of Myr-36PW against mammalian cells. **(A)** Hemolysis activity of Myr-36PW against mouse erythrocytes. **(B)** Cytotoxicity of Myr-36PW against NIH 3T3 cells from MTT assays. Results are the mean ± SD (*n* = 5).

Myr-36PW concentrations from 2.5 to 640 μg/mL were used to further detect its hemolysis and cytotoxicity activity toward mouse erythrocytes and NIH 3T3 cells. As shown in Figure 5A, Myr-36PW displayed increased hemolytic activity, and its hemolysis rate was nearly 30% at a concentration of 640 μg/mL, which was slightly higher than that of PMAP-36PW. However, no significant difference was observed at all test concentrations. The cytotoxicity results in Figure 5B were similar to those for hemolytic activity. Compared with PMAP-36PW, Myr-36PW with an added fatty acid exhibited an increase in cytotoxicity against NIH 3T3 cells, but its cell viability was above 70% even at the concentration of 640 μg/mL. Therefore, Myr-36PW remains safe for mammalian cells.

### Biofilm Inhibition and Eradication of Myr-36PW

**Figure 6:**
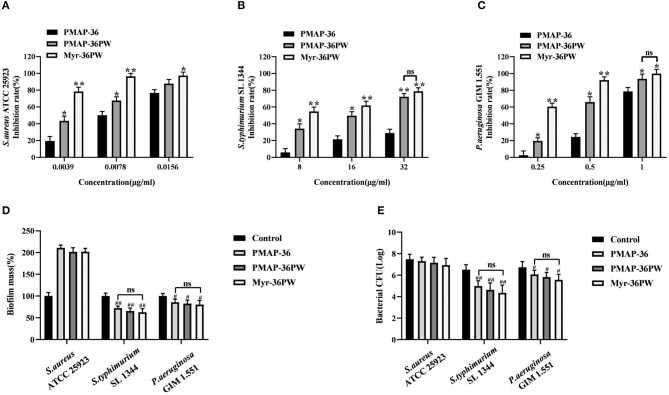
Biofilm inhibition and eradication of Myr-36PW. **(A–C)** Inhibition of biofilm formation by Myr-36PW. **(D)** Eradication of preformed biofilm by Myr-36PW. **(E)** Killing effect of Myr-36PW against biofilm bacteria. Inhibition rate % = [1 – OD_620 nm(peptides)_/OD_620 nm(bacteria)_] × 100%. Biofilm mass % = OD_620 nm(peptides)_/OD_620 nm(control)_ × 100%. The control group was bacterial culture without peptides. Data represent the mean ± SD (*n* = 5). The number of bacteria (CFU) was displayed in the form of Log. *P < 0.05 and **P < 0.01, compared with PMAP-36. ^#^*P* < 0.05 and ^*##*^*P* < 0.01, compared with control. ns denotes no significance.

The biofilm inhibition and eradication activities of Myr-36PW against *S. aureus* ATCC 25923, *S. typhimurium* SL 1344, and *P. aeruginosa* GIM 1.551 were determined via crystal violet staining to analyze its anti-biofilm activity. Myr-36PW, PMAP-36PW, and PMAP-36 generally showed biofilm inhibition against the tested bacteria in a concentration-dependent manner (Figures 6A–C). Compared with the control group, Myr-36PW (*P* < 0.01) and PMAP-36PW (*P* < 0.05) at low concentration showed significant difference in their effects against bacteria. However, Myr-36PW, PMAP-36PW, and PMAP-36 only eradicated the preformed biofilm in Gram-negative bacteria, and no significant difference was observed among the three peptides (Figure 6D). As shown in Figure 6E. Myr-36PW, PMAP-36PW, and PMAP-36 also exhibited bacterial killing effect except against *S. aureus* ATCC 25923, and this finding was consistent with the biofilm eradication results. These results showed that Myr-36PW has a selective anti-biofilm effect.

### Membrane Permeability of Myr-36PW

**Figure 7:**
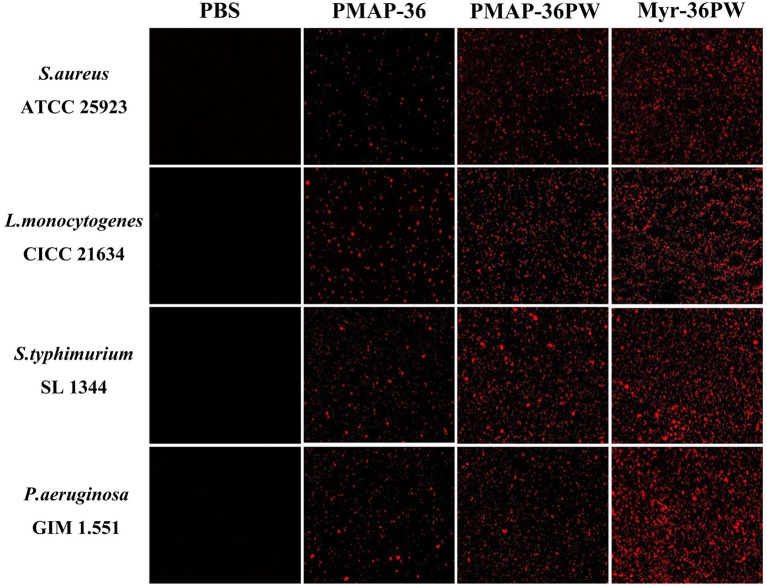
Myr-36PW membrane permeability against bacteria. The effect of Myr-36PW and its analogs on membrane permeability as evaluated by inverted fluorescence microscopy using PI as an indicator.

Membrane permeability test was performed to analyze the killing mechanism of Myr-36PW against *S. aureus* ATCC 25923, *L. monocytogenes* CICC 21634, *P. aeruginosa* GIM 1.551, and *S. typhimurium* SL 1344. Figure 7 shows that under an inverted fluorescence microscope, all PBS groups did not exhibit any red fluorescence, indicating no bacterial death. However, Myr-36PW, PMAP-36PW, and PMAP-36 groups exhibited red fluorescence against the four bacteria. However, the largest number of dead bacteria was found in the Myr-36PW groups, and the red fluorescence was distributed to almost the whole field of view. These results showed that the three peptides may kill bacteria by permeabilizing the bacterial membrane, and Myr-36PW could improve the membrane permeability of bacterial cells.

### Myr-36PW Protects Mice Against Lung Infection

**Figure 8:**
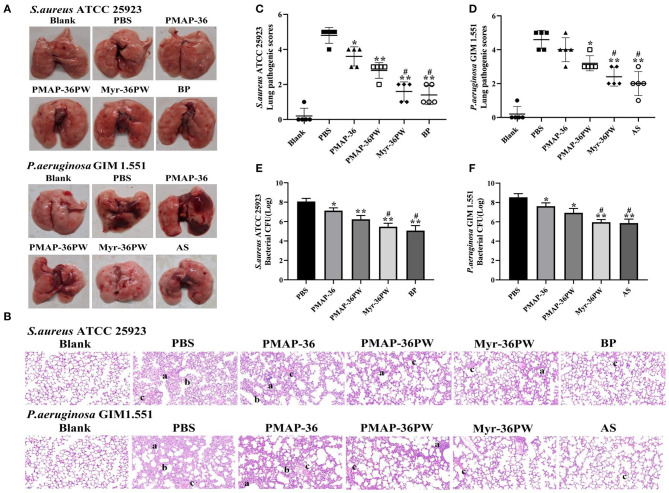
Therapeutic effect for pneumonia by Myr-36PW *in vivo*. Mice were injected with *S. aureus* ATCC 25923 or *P. aeruginosa* GIM 1.551 to form pneumonia, and lungs (*n* = 5) in each group were collected on day 4 after intranasal bacterial inoculation. **(A)** Morphological changes of lung tissues. **(B)** Representative histopathological images of processed lung section with hematoxylin and eosin (H&E) staining. Images are presented at a magnification of 20×. a: inflammatory cell infiltration. b: the alveolar wall is thickened. c: alveolar wall congestion or alveolar hemorrhage. **(C,D)** Pathological scores of the lung sections. Scoring standard: 1 indicated no pathology, 2 indicated local pulmonary congestion and inflammatory cell infiltration, 3 indicated pulmonary congestion and a large number of inflammatory cell infiltration, affecting <20% of the section, 4 indicated pulmonary congestion and a large number of inflammatory cell infiltration, affecting 20–50% of the section, and 5 indicated pulmonary congestion and a large number of inflammatory cell infiltration, affecting >50% of the section. **(E,F)** Bacterial CFU in lung homogenate. BP is benzylpenicillin potassium, AS is ampicillin sodium. Data represent the mean ± SD of five individual experiments. The number of bacteria (CFU) was displayed in the form of Log. *P < 0.05 and **P < 0.01, compared with PBS. ^#^*P* < 0.05, compared with PMAP-36PW.

The mice were intranasal inoculated with *S. aureus* ATCC 25923 and *P. aeruginosa* GIM 1.551 to induce lung disease and evaluate the therapeutic effect of Myr-36PW *in vivo*. After 3 days of treatment, anatomy results indicated no damage on the lung tissues in *S. aureus* ATCC 25923 model or local lung necrosis in *P. aeruginosa* GIM 1.551 model (Figure 8A). The lung tissues were stained with H&E to further clarify the therapeutic effect (Figure 8B). The lung tissues of PBS groups were seriously damaged with congested or thickened alveolar wall and a large number of infiltrating inflammatory cells but no normal alveolar structure. Meanwhile, Myr-36PW, benzylpenicillin potassium in *S. aureus* ATCC 25923 model, and Myr-36PW, ampicillin sodium in *P. aeruginosa* GIM 1.551 model showed a complete alveolar structure, reduced blood stasis, and large reduction in inflammatory cells. The pathology scores were recorded to reflect the differences in treatment effects. As shown in Figures 8C,D, Myr-36PW, benzylpenicillin potassium in *S. aureus* ATCC 25923 model and Myr-36PW, ampicillin sodium in *P. aeruginosa* GIM 1.551 model showed lower scores than PMAP-36PW group (*P* < 0.05). The bacterial CFU results were shown in Figures 8E,F, were similar to the pathology score results. The CFU level for *S. aureus* ATCC 25923 was reduced for PMAP-36PW, Myr-36PW and benzylpenicillin potassium group compared with that for the PBS group (*P* < 0.01), but the level of Myr-36PW and benzylpenicillin potassium group was lower than that for PMAP-36PW group (*P* < 0.05). Myr-36PW and ampicillin sodium group also showed decreased the *P. aeruginosa* GIM 1.551 CFU level compared with the PMAP-36PW group (*P* < 0.05). These results indicated that Myr-36PW is highly effective at removing bacteria and reducing pathological damage in lung tissues, with no significant difference from antibiotics.

### Therapeutic Value of Peritonitis by Myr-36PW

**Figure 9:**
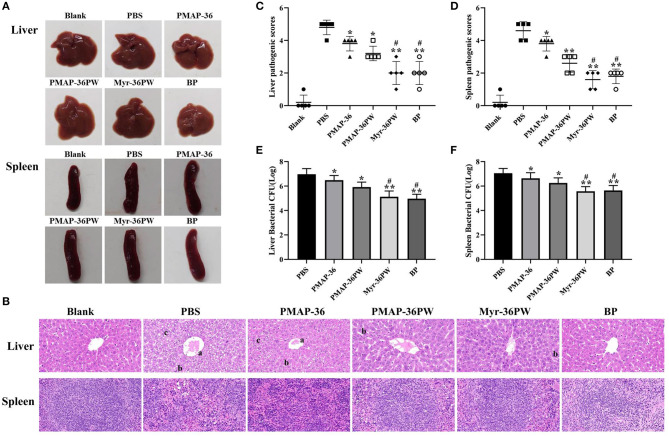
Therapeutic potency of peritonitis by Myr-36PW *in vivo*. The livers and spleens (*n* = 5) in each group were collected on day 4 after the intraperitoneal injection of *S. aureus* ATCC 25923. **(A)** Morphological changes in liver and spleen tissues. **(B)** Representative histopathological images of processed liver and spleen sections with hematoxylin and eosin (H&E) staining. Images are presented at a magnification of 40×. a: central venous hemorrhage. b: the cells fuse into pieces, and the nucleus dissolves and disappears. c: cytoplasmic cavitation. **(C,D)** Pathological scores of the liver and spleen sections. Scoring standard: 1 indicated no pathology, 2 indicated perivascular infiltrates, 3 indicated perivascular or interstitial infiltrates affecting <20% of the section, 4 indicated perivascular or interstitial infiltrates affecting 20–50% of the section, and 5 indicated perivascular or interstitial infiltrates affecting >50% of the section. **(E,F)** Bacterial CFU in liver and spleen homogenate. BP is benzylpenicillin potassium. Data represent the mean ± SD of five individual experiments. The number of bacteria (CFU) was displayed in the form of Log. *P < 0.05 and **P < 0.01, compared with PBS. ^#^*P* < 0.05, compared with PMAP-36PW.

The mice were intraperitoneally injected with *S. aureus* ATCC 25923 to further analyze the therapeutic values of Myr-36PW in peritonitis model. Figure 9A shows no damage on the liver and spleen tissues of mice, which were subsequently stained with H&E as displayed in Figure 9B. The tissues in PBS group were seriously damaged with central vein bleeding, the liver cells were arranged irregularly, the cells fused into a slice, nucleus lysis and disappearance occurred, and cytoplasmic vacuole and lymphocytes decreased in the spleen. In Myr-36PW and benzylpenicillin potassium group, the liver cells of mice were neatly arranged with clear cell boundaries and no pathological changes in spleen tissues. Statistical difference was found in pathology scores in Figures 9C,D. Compared with those for the PBS group, the liver pathogenic scores were significantly lower for PMAP-36PW (*P* < 0.05) and Myr-36PW and benzylpenicillin potassium group were significantly lower than PBS group (*P* < 0.01). Compared with those for the PBS group, the spleen pathogenic scores for PMAP-36PW, Myr-36PW and benzylpenicillin potassium groups displayed significant difference (*P* < 0.01), and those for Myr-36PW and benzylpenicillin potassium groups were lower than those for PMAP-36PW group (*P* < 0.05). The CFU number of bacterial in liver and spleen tissues was further determined for each group, and the results are shown in Figures 9E,F. The CFU levels of Myr-36PW and benzylpenicillin potassium groups in the liver and spleen were significantly decreased compared with that of PMAP-36PW group (*P* < 0.05), which was similar to the pathology scores. These results indicated that Myr-36PW could effectively inhibit bacterial growth and reduce pathological damage in liver and spleen tissues with no significant difference from antibiotics.

### Myr-36PW Promotes Mice Abscess Reduction

**Figure 10:**
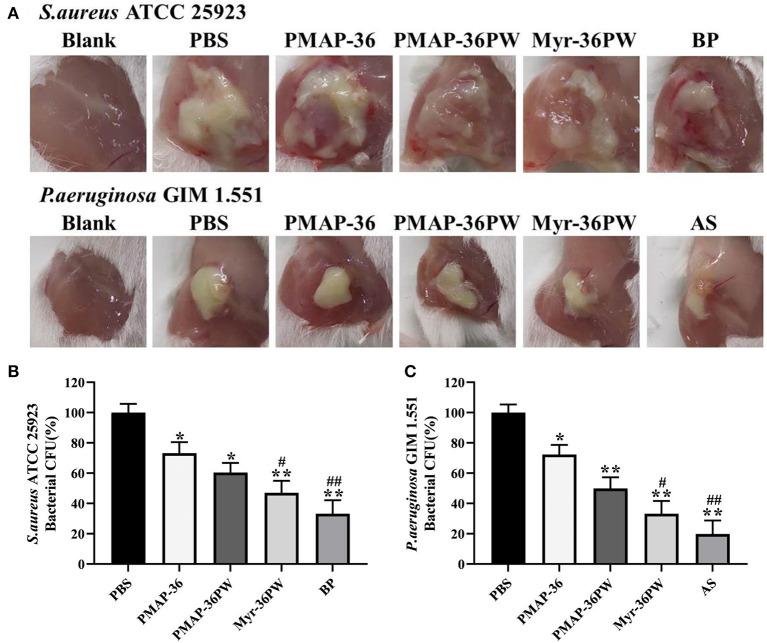
Treatment efficacy of subcutaneous abscess by Myr-36PW *in vivo*. Mice were injected with *S. aureus* ATCC 25923 or *P. aeruginosa* GIM 1.551 to develop a subcutaneous abscess before treatment. **(A)** Changes in the abscess area of mice after 3 days treatment. **(B,C)** Quantitative analysis of bacterial CFU in abscess area that received 3 days treatment under various experimental conditions. The number of CFU in each treatment group was normalized by using the CFU of the experimental group divided by the CFU of the PBS group (100%). BP is benzylpenicillin potassium, AS is ampicillin sodium. Data represent the mean ± SD of five individual experiments. *P < 0.05 and **P < 0.01, compared with PBS. ^#^*P* < 0.05 and ^*##*^*P* < 0.01, compared with PMAP-36PW.

The abscess model was also used to evaluate the therapeutic performance of Myr-36PW *in vivo*. Abscesses were formed by injecting *S. aureus* ATCC 25923 or *P. aeruginosa* GIM 1.551, and the changes were recorded and are shown in Figure 10A. A large area of abscess appeared in PBS groups, the thick juice was reduced in *S. aureus* ATCC 25923 model, and the pus volume decreased in *P. aeruginosa* GIM 1.551 model after 3 days treatment with peptides or antibiotics. In particular, the Myr-36PW, benzylpenicillin potassium in *S. aureus* ATCC 25923 model and Myr-36PW, ampicillin sodium group in *P. aeruginosa* GIM 1.551 model significantly reduced the abscess area and effectively suppressed its growth. The bacteria CFU in abscess area was further measured after the 3 day treatment, and the results are shown in Figures 10B,C. The *S. aureus* ATCC 25923 CFU level in Myr-36PW and benzylpenicillin potassium group was lower than that in PBS group (*P* < 0.01), and PMAP-36PW group showed significant difference compared with the PBS group (*P* < 0.05). The *P. aeruginosa* GIM 1.551 CFU levels of PMAP-36PW, Myr-36PW, and ampicillin sodium groups exhibited significant difference compared with that of PBS group (*P* < 0.01), but those of Myr-36PW (*P* < 0.05) and ampicillin sodium groups (*P* < 0.01) were lower than that of PMAP-36PW group. These results revealed that Myr-36PW could effectively suppress abscesses and eliminate the majority of bacteria.

### Myr-36PW Accelerates Wound Healing

**Figure 11:**
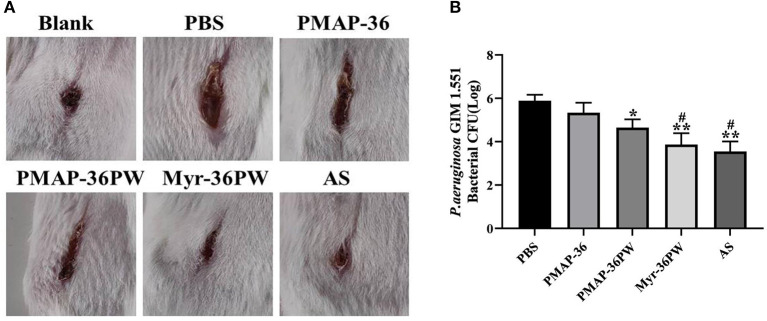
Treatment value of wound by Myr-36PW *in vivo*. A 2 cm wound was cut with a knife and directly injected with *P. aeruginosa* GIM 1.551. **(A)** Wound changes of mice after 7 days treatment. **(B)** Bacterial CFU in the wound area treated for 7 days under different experimental conditions. AS is ampicillin sodium. Data represent the mean Log (CFU) ± SD of five individual experiments. *P < 0.05 and **P < 0.01, compared with PBS. ^#^*P* < 0.05, compared with PMAP-36PW.

Mouse wound models were built to analyze the healing effect of Myr-36PW. *P. aeruginosa* GIM 1.551 was inoculated directly at the wound site, and the wound changes were recorded after 7 days of treatment (Figure 11A). The wound of blank group healed into small holes, and the mice in PBS group had wide wounds. After treatment, the width and length of the wounds in treatment groups gradually decreased. By comparison, the wounds in Myr-36PW and ampicillin sodium groups were significantly reduced. The bacterial count results were consistent with the wound changes. As Figure 11B shows that compared with that of the PBS group, the bacterial CFU was significantly reduced in the PMAP-36PW (*P* < 0.05), Myr-36PW and ampicillin sodium groups (*P* < 0.01). No significant difference was found between Myr-36PW and ampicillin sodium groups. These findings indicated that Myr-36PW can promote wound healing and reduce wound bacterial infection with comparable effects to antibiotics.

## Discussion

Bacterial resistance and antibiotic residues have become increasingly prominent, and antimicrobial resistance has become a threat to global public health systems due to the emergence of drug-resistant strains (Li et al., 2013; Penesyan et al., 2015; Ferri et al., 2017). Approximately 7,000,000 people die each year from antibiotic resistance worldwide (Willyard, 2017). Hence, new antimicrobial agents must be urgently developed. Although AMPs are promising candidates, many problems still limit their clinical use.

Fatty acids are important components of biological membrane phospholipids and have high hydrophobicity. Coupling fatty acids with AMPs can promote the membrane interaction by increasing hydrophobicity and thus enhancing the antibacterial activity (Koh et al., 2015). Fatty acids modification on some cationic peptides has been studied. Cationic amphiphilic peptide CM4 increases hydrophobicity through N-terminal myristoylation and improves its anticancer activity (Li et al., 2018). However, a previous study showed that a length-dependent relationship between antimicrobial activity and fatty acid chain (Chu-Kung et al., 2010). Therefore, the appropriate length is crucial to the antibacterial activity of AMPs. In our previous study, we designed two new peptides PMAP-36PW and PMAP-36PK by amino acid substitution. PMAP-36PW showed better antimicrobial activity *in vitro* and therapeutic potential *in vivo* than PMAP-36PK (Zhou et al., 2019b). However, PMAP-36PW still has some shortcomings: The antimicrobial activity of PMAP-36PW on Gram-negative bacteria was worse than that on Gram-positive bacteria, and PMAP-36PW showed no antimicrobial activity against *Enterococcus faecium* B21. Besides, there is still a difference between the *in vivo* therapeutic efficacy of PMAP-36PW and that of antibiotics. Studies have shown that modifying the N-teriminus can enhance the antibacterial activity of peptides, and the length of the fatty acid chain can influence the antibacterial activity and cytotoxicity of the peptides (Zhong et al., 2019, 2020). Moreover, lipopeptides have the advantages of broad-spectrum antibacterial activity, rapid bactericidal activity, and slow drug resistance tendency. Synthetic lipopeptides can effectively improve the therapeutic potential of peptides (Koh et al., 2017). Especially, cationic antimicrobial peptide HD5 modified by myristic acid can promote self-assembly of peptide to form nano antimicrobial peptide, and exhibited more efficient, rapid and broad-spectrum antibacterial activity, which is a simple and versatile strategy to generating AMP-derived nanobiotics with excellent *in vivo* tolerability (Lei et al., 2018). Thus, in the present study, we designed a new peptide Myr-36PW by coupling the myristic acid to the N-terminal of PMAP-36PW to increase its hydrophobicity. Our data also indicated that N-terminal fatty acid modification may be an effective way to develop new AMPs.

The antimicrobial activity of Myr-36PW was detected against four bacterial strains. Inconsistent with previous studies, *L. monocytogenes* CICC 21533 was replaced by *L. monocytogenes* CICC 21634 in the present work, because the MIC values of former were higher than those of other bacterial strains, and thus are not valuable for evaluating the antibacterial activity of the peptides. The four strains are two Gram-positive (*S. aureus* ATCC 25923 and *L. monocytogenes* CICC 21634) and two Gram-negative strains (*S. typhimurium* SL 1344 and *P. aeruginosa* GIM 1.551). Meanwhile, they are all zoonotic pathogens, and cause serious harm to animal and human health. In addition, the MICs of bacteria to different classes of antibiotics were analyzed according to the CLSI criteria, and the results showed that four strains exhibited different degrees of resistance (Supplementary Table 3). Two of them are drug-resistant strains (*S. aureus* ATCC 25923 and *S. typhimurium* SL 1344), and the other two are multi drug-resistant strains (*L. monocytogenes* CICC 21634 and *P. aeruginosa* GIM 1.551). The antimicrobial activity results showed that Myr-36PW exhibited excellent antimicrobial activity higher than PMAP-36PW, suggesting that hydrophobic fatty acids play an important role in the antimicrobial activity. In addition, the MIC values of Myr-36PW against Gram-positive and Gram-negative bacteria were decreased compared with those of PMAP-36PW. PMAP-36PW itself effectively killed both types of bacteria *in vitro*, but Myr-36PW exhibited potential bactericidal activity compared with PMAP-36PW. The current modification strategy did not change the arrangement of the hydrophilic and hydrophobic amino acids of the parent peptide PMAP-36PW. The new peptide Myr-36PW maintained the original positive charge, and the combination of fatty acids increased its hydrophobicity and enhanced its antibacterial activity, which is consistent with previous studies (Malina and Shai, 2005; Krishnakumari et al., 2018; Zhong et al., 2020). In addition, fatty acid addition can improve the hydrophobicity and promote the folding or aggregation of AMPs in the solution (Avrahami and Shai, 2002; Chu-Kung et al., 2004). As a cationic antimicrobial peptide, Myr-36PW with N-terminal myristoylation exhibited increased hydrophobicity but not self-assembly, possibly due to the high cationic peptide segment found at the N-terminal of PMAP-36PW, that is disadvantageous to self-assembling micelles based on intermolecular interactions.

AMPs are susceptible to proteolytic degradation *in vivo* because their bactericidal activity in body fluids can be negated by pH, salts, or serum that can interfere through non-specific electrostatic interactions (Svenson et al., 2007). Thus, the stability of Myr-36PW exposed to heat, pH, salts, and serum was researched. Thermal and pH stability tests indicated that Myr-36PW was more stable than PMAP-36PW. The antimicrobial activity of Myr-36PW against *P. aeruginosa* GIM 1.551 and *S. aureus* ATCC 25923 remained more stable than that of PMAP-36PW after boiling for 120 min and within a wide pH range. Salt ions and serum reduce the antibacterial activity of antibacterial peptides (Walkenhorst, 2016; Zhong et al., 2019). In this study, physiological activity salts or serum environment also changed the MICs values of Myr-36PW, but no loss of antimicrobial activity was observed. In addition, NaCL and CaCL_2_ have different effects on the antimicrobial activity of Myr-36PW, the physiological concentration of divalent-cation leads to the high affinity of Myr-36PW to bacterial cell wall and consequent great antimicrobial activities (Hong et al., 2017). Thus, these findings indicated the antimicrobial peptide Myr-36PW with N-terminal myristolyation exhibited thermal and pH stabilities and salt or serum resistance.

An excellent antimicrobial peptide for clinical application must show efficient, rapid, and stable antibacterial activity and low toxicity to mammalian cells. In general, hemolysis and cytotoxicity are positively correlated with the hydrophobicity of peptides; the increase in the hydrophobicity of peptides could enhance the selectivity, but a further increase could produce negative influence (Dong et al., 2014; Schmidtchen et al., 2014; Zhong et al., 2020). The toxicity of Myr-36PW to erythrocytes and NIH 3T3 cell increased after N-terminal myristolyation. At the concentration of 640 μg/mL, Myr-36PW displayed ~30% hemolytic activity and 70% cell survival, which were slightly higher than those of PMAP-36 and PMAP-36PW. After fatty acid modification, Myr-36PW exhibits increased hydrophobicity and enhanced cytotoxicity to normal mammalian cells but still showed considerable biosecurity.

Antibiotics and other antimicrobial products cannot eliminate bacterial biofilm because only 0.1% of the microbial population actually grow in planktonic mode, resulting in the development of bacterial resistance (Bjarnsholt et al., 2013; de Breij et al., 2018). Prevent biofilm formation or treating established biofilms by using AMPs has become a research focus (Dosler and Karaaslan, 2014; Chen et al., 2018; Mwangi et al., 2019b). In our study, we further analyzed the anti-biofilm effect of peptides by using two bacteria with strong biofilm (*S. aureus* ATCC 25923 and *P. aeruginosa* GIM 1.551) and one bacterial strain with weak biofilm (*S. typhimurium* SL 1344). Compared with PMAP-36, Myr-36PW could significantly inhibit the biofilm formation of three bacteria. Myr-36PW also showed biofilm eradication but only for Gram-negative bacteria without any effect on *S. aureus* ATCC 25923. No significant difference was observed among the three peptides possibly because lipopolysaccharide (LPS) is the major molecular component of the outer membrane of Gram-negative bacteria, and AMPs have a high affinity to LPS (Rosenfeld and Shai, 2006). In this study, the bacteria killing mechanism was also investigated via membrane permeability assay. AMPs first interact with anionic bacterial membranes through electrostatic interactions, and hydrophobic parts are inserted into the phospholipid layer to destroy the membrane structure (Edwards et al., 2016). The hydrophobicity of Myr-36PW increased significantly after binding with myristic acid and showed strong membrane permeability, thus increasing bacterial killing. Modification with fatty acids such as myristic acid can promote peptide membrane interactions, provide an anchor for fatty acyl chains that are inserted into the bacterial membrane, and result in enhanced membrane permeability and eventually bacterial killing (Lockwood et al., 2004; Krishnakumari et al., 2018).

Myr-36PW antibacterial activity *in vivo* was further investigated due to its excellent antibacterial activity and good stability *in vitro*. Pneumonia, peritonitis, subcutaneous abscesses and wound infection models were built to evaluate the therapeutic efficacy of this peptide *in vivo*. As the most common clinical drug-resistant bacteria to cause localized and systemic infection, *P. aeruginosa* GIM 1.551 and *S. aureus* ATCC 25923 strains were used to infect mice (van Delden, 2007; Deleon et al., 2014; Santajit and Indrawattana, 2016). In the pneumonia and peritonitis models, PMAP-36 and PMAP-36PW had poor therapeutic effect *in vivo*, but Myr-36PW significantly decreased the following: bacterial CFU to target organs, including lung, liver, and spleen; tissue damages and inflammatory cell infiltration; and pathological scores. These results indicated the antimicrobial and anti-inflammatory of Myr-36PW *in vivo*. The therapeutic efficacy of Myr-36PW was also highlighted in subcutaneous abscesses and wound infection models. Myr-36PW could better suppress bacterial growth at the target site and promote abscess reduction and wound healing compared with the same dose of PMAP-36 and PMAP-36PW. It has been reported that antimicrobial peptides can mediate inflammation and improve wound healing (Koczulla and Bals, 2003; Sebe et al., 2016). Moreover, the antimicrobial peptide Nano-BA_5k_ promotes wound healing by decreasing bacterial counts of wound, which was consistent with our results that antimicrobial peptides can improve wound healing by reducing the number of bacteria in infected wounds (Hong et al., 2017). Certainly, wound healing also involves inflammation, re-epithelialization, angiogenesis, granulation tissue formation and other physiological events. And granulation tissue formation plays a key role in wound healing (Murthy et al., 2013). Whether Myr-36PW improves wound healing in other ways requires further study. This finding further revealed that N-terminal myristoylation is an effective modification strategy, and Myr-36PW is a promising anti-infective agent for the treatment of bacterial infections.

In conclusion, our results revealed the potentials of Myr-36PW, an N-terminal fatty acid modification peptide, and a promising drug candidate with good stability and considerable toxicity and can enhance antimicrobial activity, exhibit anti-biofilm activity, and kill bacteria by permeabilizing the bacterial membrane. Myr-36PW also showed an impressive therapeutic effect in *S. aureus* ATCC 25923 and *P. aeruginosa* GIM 1.551 induced infection *in vivo*. This study can serve as a reference for developing promising drug candidates against antibiotic resistance.

## Data Availability Statement

All datasets generated for this study are included in the article/Supplementary Material.

## Ethics Statement

The animal study was reviewed and approved by Animal Experiment Committee of Henan University of Science and Technology.

## Author Contributions

CW and CL designed the study. TS and SS performed experiments. LC and JZ performed data analysis. YW and ZZ provided materials or resources. YL and SL wrote the draft. YL, CW, and CL revised the manuscript. All authors read and approved the final manuscript.

## Conflict of Interest

The authors declare that the research was conducted in the absence of any commercial or financial relationships that could be construed as a potential conflict of interest. The reviewer FS declared a shared affiliation with one of the authors, SL to the handling editor at time of review.
