# Supplementary material for: N-terminal Myristoylation Enhanced the Antimicrobial Activity of Antimicrobial Peptide PMAP-36PW
Source: Front Cell Infect Microbiol. 2020 Aug 27;10:450. doi: 10.3389/fcimb.2020.00450 (PMC7481357; doi:10.3389/fcimb.2020.00450)
Supplement: Supplementary file 1 [file Data_Sheet_1.docx]

***Supplementary Material***


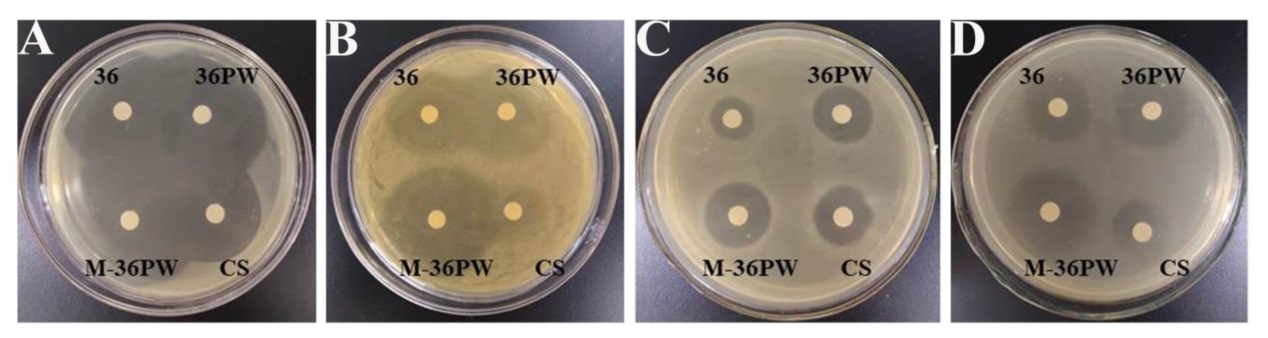


**Supplementary Figure 1** Inhibition zone of Myr-36PW. The antibacterial susceptibility test of Myr-36PW and its analogs against different bacteria was performed using disk diffusion method. **(A)** *S. aureus* ATCC 25923 **(B)** *L. monocytogenes* CICC 21634 **(C)** *S. typhimurium* SL 1344 **(D)** *P. aeruginosa* GIM 1.551 (CS) Ceftiofur sodium. The diameter of the standard disk is 6 mm.

**Supplementary Table 1** *In vitro* antibacterial activity of Myr-36PW

| **Bacteria strain** | **PMAP-36** | **PMAP-36PW** | **Myr-36PW** | **Ceftiofur Sodium** |
| --- | --- | --- | --- | --- |
| ***S. aureus* ATCC 25923** | **+** | **+** | **+**** | **+** |
| ***L. monocytogenes* CICC 21634** | **+** | **+** | **+**** | **+** |
| ***S. typhimurium* SL 1344** | **+** | **+** | **+*** | **+** |
| ***P. aeruginosa* GIM 1.551** | **+** | **+** | **+**** | **+** |

The *in vitro* antibacterial activity was determined by disk diffusion method. +, means the peptide has antibacterial activity. *, *P* < 0.05 and **, *P* < 0.01, compared with the PMAP-36PW.

**Supplementary Table 2** Salt and serum stability of Myr-36PW

| **Bacteria strain** | **Peptide** | **MICs(μg/mL)** | | | |
| --- | --- | --- | --- | --- | --- |
|  |  | **Control** | **NaCl** | **CaCl_2_** | **Serum** |
| ***S. aureus* ATCC 25923** | **PMAP-36** | 0.0625 | 0.0625 | 0.0625 | 0.0625 |
|  | **PMAP-36PW** | 0.0313 | 0.0313 | 0.0313 | 0.0313 |
|  | **Myr-36PW** | 0.0156 | 0.0156 | 0.0078 | 0.0313 |
| ***P. aeruginosa* GIM 1.551** | **PMAP-36** | 2 | 2 | 2 | 2 |
|  | **PMAP-36PW** | 1 | 1 | 1 | 1 |
|  | **Myr-36PW** | 0.25 | 0.25 | 0.5 | 0.5 |

Salt and serum stability *in vitro* were indicated by MIC assay. The concentrations of NaCl and CaCl_2_ were physiological concentration. 10% mice serum was inactivated by heat treatment for 15 min at 60 ℃。The control group was measured in the absence of physiological salts or serum in MHB.

**Supplementary Table 3** MIC of different classes of antibiotics

| Bacteria strain | MICs (μg/mL)^&^ | | | | | | | |  | |
| --- | --- | --- | --- | --- | --- | --- | --- | --- | --- | --- |
|  | Ceftiofur Sodium | Benzylpenicillin potassium | Ampicillin sodium | Azithromycin | Gentamicin | Amikacin | Colistin | Polymyxin B | |  |
| *S. aureus* ATCC 25923 | <0.25 | <0.25 | 0.5 | 64* | 4 | 4 | 16* | 16* | |  |
| *L. monocytogenes* CICC 21634 | 32* | 64 | 64 | 64* | 32* | 128* | 8 | 8 | |  |
| *S. typhimurium* SL 1344 | 4 | 16 | 16 | >128* | 8 | 32 | 2 | 2 | |  |
| *P. aeruginosa* GIM 1.551 | 64* | 64 | 32 | 128* | 128* | >128* | 4* | 4* | |  |

&, MICs (Minimum inhibitory concentrations) were determined as the lowest concentration of the antibiotics that inhibited bacteria growth. Drug-resistance was determined according to the recommendations of the Clinical Laboratory Standards Institute (CLSI, 2020). Antibiotics are classified as follows: Ceftiofur sodium, Benzylpenicillin potassium and Ampicillin sodium are β-lactam antibiotics. Azithromycin is macrolide antibiotics. Gentamicin and Amikacin are aminoglycoside antibiotics. Colistin and Polymyxin B are polypeptide antibiotics. * indicates resistant.
